# The research progress of wearable digital health technologies in epilepsy management

**DOI:** 10.1186/s42494-026-00247-5

**Published:** 2026-03-02

**Authors:** Xinyi Zhao, Tiancheng Wang

**Affiliations:** Department of Neurology, Epilepsy Center, The Second Hospital & Clinical Medical School, Lanzhou University, 82 Cuiyingmen, Chengguan District, Lanzhou City, Gansu Province 730030 China

**Keywords:** Epilepsy, Wearable technology, Epilepsy management, Artificial intelligence

## Abstract

Epilepsy is one of the most common neurological disorders, characterized by recurrent, unpredictable seizures. Due to the unpredictability of seizures, epilepsy presents unique challenges in monitoring and management. While video electroencephalogram (EEG) monitoring is the gold standard for diagnosing epilepsy, its application is limited to clinical settings and is not suitable for long-term monitoring in daily life. In recent years, the development of wearable digital health technologies has provided new solutions for epilepsy management. These technologies, utilizing artificial intelligence algorithms, can monitor the physiological state of epilepsy patients in real time, predict and record seizures, thereby optimizing the management and response to seizures, reducing injuries, and potentially lowering the risk of sudden unexpected death in epilepsy (SUDEP). This article reviews the current applications and challenges of wearable technology in epilepsy monitoring and management, and explores the future directions of its development in epilepsy care, aiming to provide insights for effective monitoring and prevention.

## Background

Epilepsy is one of the most common neurological disorders,characterized by recurrent seizures, and it affects approximately 50 million people worldwide [1]. Around 30% of epilepsy cases are resistant to treatment with antiseizure medications [2]. The paroxysmal and unpredictable nature of seizures is the main cause of suffering, morbidity, and mortality among people with epilepsy [3]. For individuals with epilepsy, as well as their families and caregivers, epilepsy is an unpredictable, challenging, and often frightening condition, especially for the one-third of patients who continue to experience seizures despite receiving treatment [4].

Changes in the frequency and severity of seizures may increase the risk of injury during daily activities or lead to status epilepticus (SE) [5] and sudden unexpected death in epilepsy (SUDEP) [6]. Epilepsy-related injuries and accidents are often associated with generalized tonic–clonic seizures (GTCS) [7]. The primary risk factor for SUDEP is increased occurrence of GTCS [8], but risk may vary depending on living conditions. People living alone are at the highest risk of frequent tonic–clonic seizures [9], and living alone or in a household without sharing a bedroom increases the risk of sudden death. For those living alone, even a convulsive seizure may go unnoticed, particularly if the seizure occurs during sleep. For children with epilepsy, parental reports may not be entirely reliable, with up to 50% of seizures going unrecognized [10]. If an individual with epilepsy is driving or working in a hazardous environment, it can endanger both the patient and others. Inaccuracies in clinical history and epilepsy reports may also lead to overdiagnosis, resulting in unnecessary treatments, work restrictions, and a decline in quality of life.

The frequency and severity of seizures vary from person to person [11]. In the case of unpredictable seizures, individuals with epilepsy may be unable to control their health condition. Currently, the gold standard for diagnosing epilepsy is video-electroencephalographic (EEG) monitoring, which includes scalp electroencephalogram (sEEG) or intracranial electroencephalogram (iEEG) [12]. These recordings are typically performed in facilities within epilepsy monitoring units, but it is difficult to monitor seizures over long periods in daily life [13]. Therefore, seizure detection devices (SDDs) and wearable digital health technologies (DHTs), which utilize artificial intelligence (AI) technologies to monitor epilepsy patients in real time, improve the quality of care for seizures, reduce injuries, and lower the risk of SUDEP, provide valuable tools for patients, caregivers, and clinicians [14].

## Methods for literature search and selection

To ensure a comprehensive, reproducible, and unbiased overview of the current research landscape, the literature search and selection for this narrative review were guided by the fundamental principles of the Preferred Reporting Items for Systematic Reviews and Meta-Analyses (PRISMA) framework. A systematic search was conducted to identify relevant publications spanning from the early foundational studies to the most recent advancements.

The electronic databases PubMed/MEDLINE, Web of Science, and IEEE Xplore were queried for literature published up to May 2025 to capture the latest developments. The search strategy employed a combination of keywords and Medical Subject Headings (MeSH) terms centered on the core concepts: ("epilepsy" OR "seizure") AND ("wearable technology" OR "digital health" OR "smartwatch" OR "accelerometer" OR "electrodermal activity" OR "electroencephalography") AND ("detection" OR "prediction" OR "monitoring" OR "management").

Inclusion criteria encompassed: (1) original research articles, systematic reviews, meta-analyses, and clinical practice guidelines; (2) studies focusing on human subjects with epilepsy or relevant caregiver/clinician perspectives; (3) literature primarily describing or evaluating wearable digital health technologies for seizure detection, prediction, or management.

Exclusion criteria were: (1) studies not published in English; (2) editorials, letters, or case reports with a sample size of less than 5 (unless highly illustrative of a novel technology); (3) articles where the full text was unavailable; (4) studies on technologies not intended for patient-worn use (e.g., stationary cameras without wearable components).

**Fig. 1:**
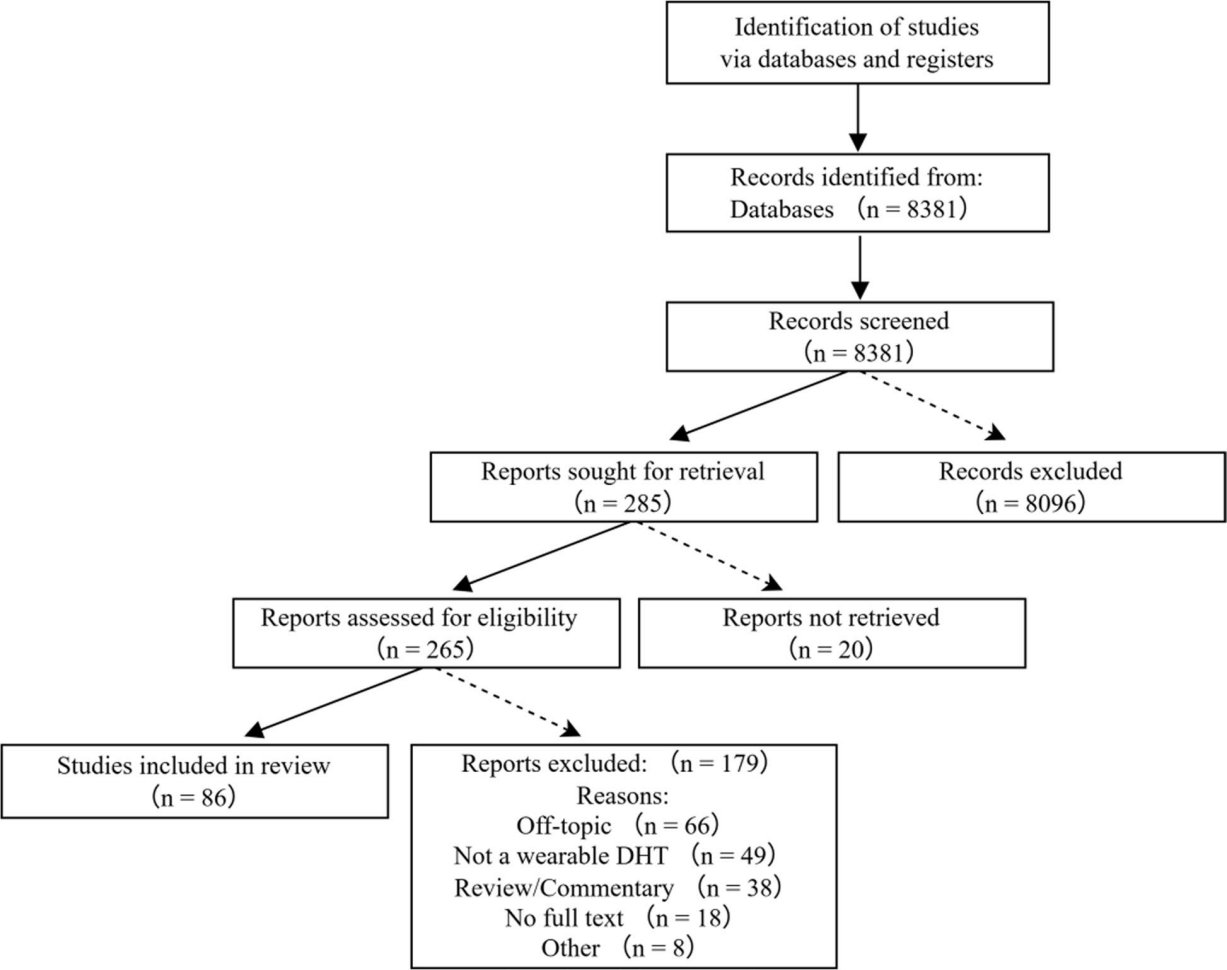
PRISMA flow diagram illustrating the literature search and selection process. The flowchart details the process of identifying, screening, assessing for eligibility, and including studies in the current narrative review

The study selection process involved multiple phases. Initially, all identified records were screened by title and abstract to remove duplicates and obviously irrelevant studies. The full texts of the remaining articles were then critically assessed for eligibility based on the predefined criteria. This rigorous process, summarized in Fig. 1, resulted in the final inclusion of 86 key publications that form the evidence base for this review. This methodology ensures that the subsequent synthesis and discussion are grounded in a robust, transparent, and representative body of literature, covering a timeline from foundational works to the most recent expert insights.

Based on the systematic literature search detailed above, this article reviews the current applications and challenges of wearable technology in epilepsy monitoring and management, and explores the future directions of its development in epilepsy care, aiming to provide insights for effective monitoring and prevention.

## Monitoring mechanisms and device types: a conceptual framework

### Monitoring mechanisms

The detection mechanisms of epilepsy monitoring devices can be broadly categorized based on the physiological signals they capture [14, 15]:

Movement and Muscle Activity Detectors: These include accelerometers (measuring body acceleration), magnetometers, piezoelectric bed sensors (detecting vibrations associated with movements), surface electromyography (sEMG; measuring muscle electrical activity), eye-tracking, and video monitors.

Autonomic Nervous System Change Detectors: These monitor alterations in bodily functions controlled by the autonomic nervous system during seizures. Key parameters include heart rate and pulse (via ECG or PPG), blood pressure, skin conductance (electrodermal activity, EDA), temperature, respiration, and oxygen saturation.

Direct Brain Activity Monitors: Electroencephalography remains the direct method for capturing cerebral electrical activity associated with seizures.

Other Mechanisms: This category encompasses audio detection of seizure-related sounds and near-infrared spectroscopy (NIRS), among other emerging sensing modalities.

**Fig. 2:**
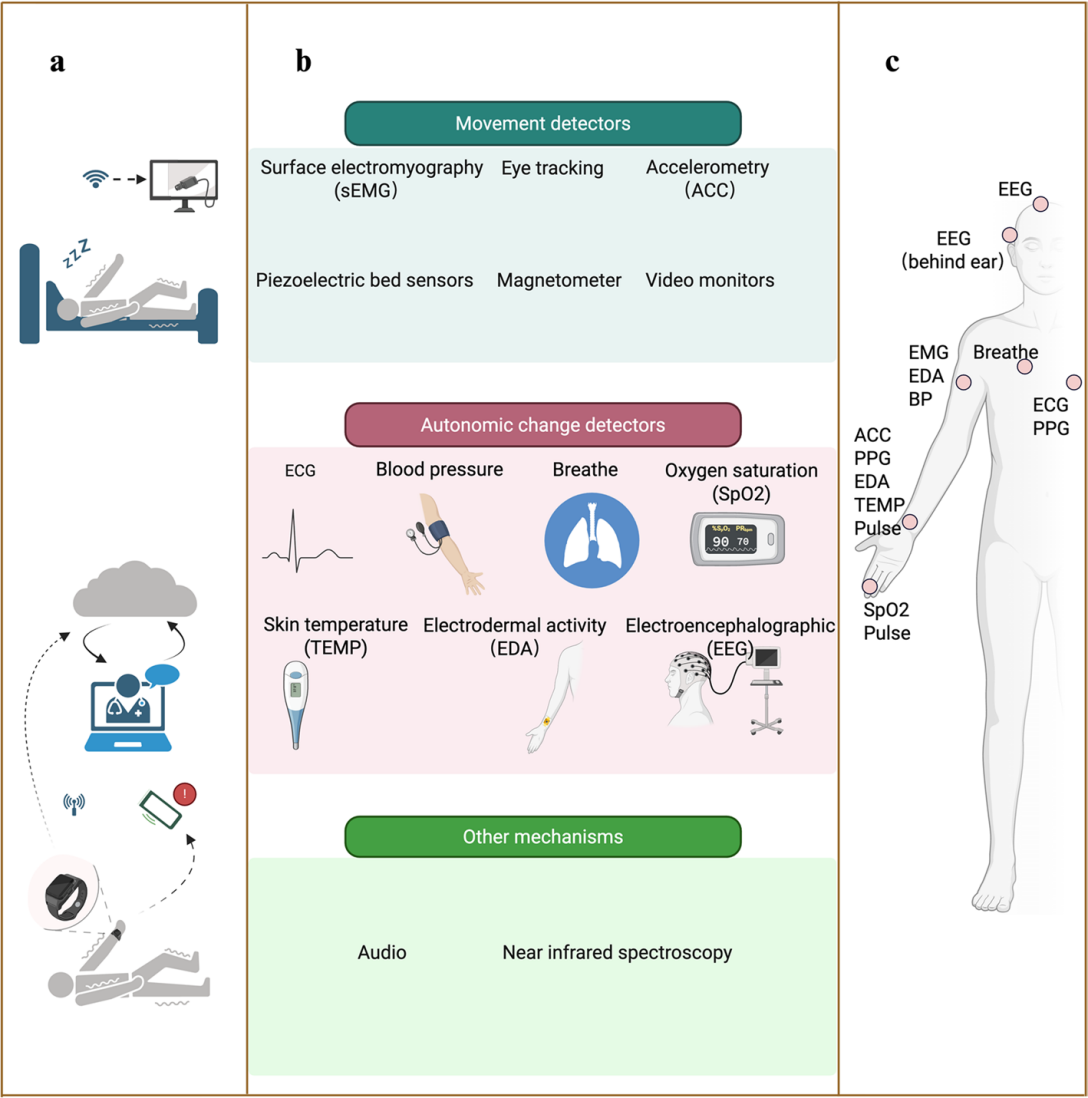
A conceptual framework for wearable digital health technologies in epilepsy management. **a** Categorization of device types by primary use case. **b** Classification of detection mechanisms by physiological signal. **c** Common wearing locations for mobile sensors. Created in BioRender. Norman, E. (2025) https://BioRender.com/

These mechanisms can be deployed individually or, increasingly, combined into multimodal wearable devices to enhance sensitivity and specificity by capturing complementary physiological signatures of epileptic events [16, 17]. The interrelationship between these mechanisms and the device types that host them is illustrated in Fig. 2.

### Types of devices for epilepsy monitoring

Digital health technology (DHT) is a system that utilizes computing platforms, connected software, and/or sensors for healthcare and related purposes [18]. Wearable DHT electronic devices include smartwatches, smart glasses, activity trackers, and implants, equipped with sensors to detect, analyze, and transmit data such as vital signs, environmental information, and biofeedback. These devices can individually or collectively assess various aspects of neurological health, including motor function, sleep, cognition, speech, EEG, pupil and eye movements, and other domains [19]. They have the potential to address longstanding gaps in continuous remote monitoring, particularly in seizure detection and prediction [20], and have become an integral part of daily life.

Currently, several epilepsy detection devices have received regulatory approval in the United States or Europe, with peer-reviewed publications reporting their performance data [16]. For specific epilepsy patients, the optimal device depends on its primary use, personal preference, age, and epilepsy type. Based on their primary use case and portability, these options can be categorized into two major types: devices designed for indoor monitoring and those intended for mobile detection.

#### Indoor monitoring devices

Indoor monitoring devices typically use non-portable mechanisms (e.g., video sensors or bed motion sensors) or non-portable control units with a limited sensor range. These devices are particularly suitable for detecting nocturnal seizures, with one of their primary objectives being to alert family members or caregivers to nighttime seizures, thereby reducing the risk of SUDEP. Home-based detection devices can be used across a broad age range, with some even suitable for infants, such as video sensors, bed motion sensors, or heart rate monitoring sensors.

#### Mobile detection devices

Mobile detection devices are wearable systems designed for use throughout the day and across different environments. They typically require a compatible smartphone as a control unit, making them portable. Some of these devices need an internet connection to send remote alerts to caregivers. These devices aim to detect seizures at any time and place, making them ideal for individuals who are more independent, may occasionally be alone, and wish to notify designated contacts when a seizure occurs. Some devices can even send a global positioning system (GPS) location via a smartphone application.

Additionally, they can track the frequency of seizures, which is particularly useful for maintaining an accurate seizure count. This data can help clinicians guide medical decisions or assist research teams in evaluating clinical trial outcomes. Wearable devices and mobile health applications have the potential to optimize personalized epilepsy care [21]. Consumer electronics, such as smartphones and smartwatches equipped with accelerometers or heart rate sensors, have already been used by epilepsy patients. However, mobile detection devices are typically designed for adults and may not be suitable for young children or infants.

While traditional EEG-based DHT devices—encompassing both scalp-EEG wearables and more invasive subcutaneous EEG systems—are commonly used in research and may offer additional features, they are often characterized by lower convenience, comfort, and aesthetic appeal [22, 23]. A key advantage of these systems is their ability to provide objective and detailed seizure characterization, offering a more accurate alternative to self-reported diaries. The current literature adequately describes their limitations. In clinical trials for new therapies, wearable DHTs can be used for objective seizure data assessment [16, 24]. Beyond seizure detection, DHTs can also collect objective data to monitor treatment-related side effects, as well as coexisting neurological, behavioral, and medical conditions associated with epilepsy.

## Performance of wearable devices for epilepsy detection

This section critically evaluates the reported performance of wearable devices, explicitly linking potential benefits to specific study populations, conditions of use, and measured effect sizes (e.g., sensitivity, false alarm rate) to enable a thorough discussion of the evidence base and its limitations.

### Identification, alerts, and localization

Wrist-worn DHT Devices: These devices typically use accelerometry and electrodermal activity (EDA) to detect patient seizures while maintaining a bluetooth connection with the device. The accompanying application transmits data and detection results to a cloud server and sends seizure alerts to caregivers [25]. Some devices also include the wearer's GPS location.

Wearable sEMG Monitoring System: This system connects through adhesive patches attached to the biceps and detects convulsions by identifying changes in electromyography (EMG) signals. The device also features a cloud-based data platform that can send alerts to caregivers [26]. Detecting convulsive or motor seizures is relatively easier compared to other seizure types [27]. Proper placement of the monitoring system is crucial for accuracy, and for some patients, minimizing false positives may be challenging.

Currently, both types of wearable sensors have been approved by the FDA and the European Union for detecting convulsive seizures. Studies report that wrist-worn accelerometry-based and surface EMG devices exhibit high sensitivity, but they also have a high false alarm rate [28]. Furthermore, sensitivity and specificity vary depending on seizure type, patient body type, skin tone, and whether the patient is at rest or engaged in physical activity during a seizure. The physiological measurement methods also differ based on these factors [29].

### Sensitivity and false alarm rate

A study compared the performance data of six wearable DHT devices sold in the U.S. or Europe with audiovisual monitoring and EEG [16]. These devices included wrist-worn accelerometers or multimodal sensors, armbands that record movement and pulse, and sEMG-based sensors. The sensitivity for detecting convulsive seizures ranged from 76 to 95%, with most devices detecting over 90% of generalized convulsive seizures when correctly placed. A systematic review and meta-analysis was conducted on the automatic detection of seizures using non-invasive wearable devices, which used video EEG monitoring as the gold standard to determine the sensitivity and false alarm rate (FAR) of non-invasive wearable devices. The results indicated that wearable devices had high sensitivity for detecting generalized tonic–clonic seizures within the limited recording time in the video EEG environment, but with a relatively high FAR. Future studies should focus on reducing FAR, detecting other seizure types, psychogenic non-epileptic seizures (PNES), and conducting longer duration recordings in community settings [28].

Postauricular EEG: A study using postauricular EEG for recording focal seizures found a sensitivity of 96.3%, with a false detection rate of 0.14 per hour. This study paved the way for the development of wearable devices for postauricular EEG monitoring [30].

Multimodal Sensors: Research has shown that multimodal sensors exhibit better sensitivity than single sensors [31]. In a multicenter, home-based, prospective, video-controlled cohort study, detecting nocturnal seizures in epilepsy and intellectual disability patients through heart rate (using PPG) or movement (using 3D accelerometry) suggested that combining heart rate and movement could reliably detect a wide range of nocturnal seizures [32]. Multimodal sensors can also provide data on the severity of seizures, with studies showing that increased heart rate and the duration of this tachycardia correlate with the subjective clinical assessment of seizure urgency [33]. One study indicated that a model well-trained in a single modality (e.g., intracranial EEG, iEEG) can be effectively generalized to a distinct modality (e.g., wearable devices) with only minimal fine-tuning using training data from the latter [34].

Machine Learning Algorithms: The use of machine learning combined with wrist accelerometers (ACM) and electrodermal activity has been shown to effectively detect both primary and secondary generalized tonic–clonic seizures. A prospective study was conducted based on predefined machine learning algorithms for GTCS detection devices using ACM and EDA, which were approved by the FDA. For commercially available DHT devices with published data, the FAR ranges from 0.1 to 2.5 times per day. The FAR is higher in children than in adults, and most false alarms occur during wakefulness [35]. The significant heterogeneity in FAR suggests that sensor types, detection algorithms, as well as the duration, usage patterns, and user-specific factors, are key to minimizing false alarms [28]. An important consideration is the wearer's ability to quickly cancel false alarms, as rapid cancellation can significantly reduce the challenge of false alarms. Some studies have demonstrated that wearable DHT devices can distinguish between epileptic and non-epileptic events, as well as detect other types of motor seizures, while further reducing FAR without compromising the sensitivity of detection algorithms [36].

Detection of Different Types of Seizures: Seizures with significant movement, such as tonic, hyperkinetic, or myoclonic seizures, may be more difficult to detect because they usually last shorter than GTCS [18]. However, wrist-worn accelerometer and pulse armband sensors have a median sensitivity of 73% to 89% for detecting these types of seizures [32]. Focal seizures, due to their highly heterogeneous clinical presentation, are being studied using EEG sensors or peripheral signals like pulse. Detection of focal motor seizures through wearable device data is possible for individuals [37]. New clinical trials for drug-resistant focal seizures will benefit from wearable devices capable of detecting the most common non-convulsive seizures. The International League Against Epilepsy (ILAE) and the International Federation of Clinical Neurophysiology (IFCN) clinical practice guidelines recommend the use of wearable devices for automatic detection of seizures, but only for GTCS and focal-to-bilateral tonic–clonic seizures (FBTCS). Further research and development are needed to improve the performance of automatic seizure detection for other seizure types, along with the accuracy and clinical utility of these devices [38].

Seizure Prediction: The goal of seizure detection is to provide an alert at the onset of a seizure, which can assist in localizing the seizure. In contrast, seizure prediction aims to forecast the impending occurrence of a seizure [39], enabling both localization and preemptive pharmacological or neurostimulation interventions. Early machine learning algorithms used for seizure prediction included logistic regression, support vector classifiers, and convolutional neural network classifiers [40]. More recently developed seizure prediction algorithms are almost entirely based on deep learning approaches [41, 42]. Closed-loop systems are being developed to detect seizures, trigger treatment responses, and even predict and prevent seizures, potentially changing the management of epilepsy [23]. Wearable DHT devices may also help estimate the risk of SUDEP. Data recorded by these devices assist in personalized risk calculation, deepen our understanding of the mechanisms of SUDEP in epilepsy patients, and provide valuable information for preventive interventions [43]. A study surveyed epilepsy patients and caregivers about visual designs for seizure prediction, finding that radar charts, line graphs, and heatmaps were the three most popular options for providing a software interface on open communication platforms for patients and clinicians [44]. By tracking time periods [45], physiological signals [46], or combinations thereof, it is possible to non-invasively predict seizures, which has proven transformative for many patients. These challenges and the complexity of accurately forecasting seizures have prompted the adoption of deep learning approaches for seizure prediction.

In addition to sensing basic biological signals, wearable devices and smartphones (including apps) can also track more complex behaviors such as activity patterns, range of motion, sleep duration and quality, and emotional and behavioral indicators, such as analysis based on mobility speed, social connections, or emotional tone [47]. Furthermore, there is emerging recognition of the potential for these wearable technologies to monitor circadian and multi-day rhythms. Evidence indicates that over 90% of people with epilepsy exhibit circadian rhythms in their seizure occurrence, and many also experience multiday cycles. Analyzing these inherent rhythms enables the forecasting of seizure susceptibility, representing a promising approach for seizure prediction [48].

## Willingness to use digital technologies and wearable devices & influencing factors

### Acceptability

One key aspect of wearable technology adoption is its acceptability in terms of design. Multiple studies have explored the perspectives of epilepsy patients and their caregivers on various aspects of wearable devices [49]. These devices help patients communicate with doctors about seizure occurrences, enhance self-management, raise awareness, improve activity planning, and increase safety. However, the main barriers include potential stigma and anxiety. Stigma associated with epilepsy is a significant global issue that affects patients' quality of life [50, 51]. Familiar and customizable products may be important moderating factors influencing participation. Studies assessing user experience have shown higher acceptance of smartphone applications compared to standalone wearables [52, 53].

### Engagement

While mobile health (mHealth) tools offer real-time measurement and management capabilities, their effectiveness relies on user engagement. Long-term engagement is critical for successfully adopting eHealth technologies, especially for chronic conditions like epilepsy. Engagement is defined by how actively individuals use these resources, such as wearing the device and interacting with smartphone applications [54]. Factors influencing engagement are complex and multifactorial, often shaped by cultural backgrounds and personal experiences. The unpredictability of seizures may affect users' willingness to engage with these tools consistently. Beyond health monitoring, technologies that integrate multiple service functions into daily life may provide additional advantages in sustaining user engagement.

### Cost, usability, comfort, and data accuracy

Several key factors determine whether epilepsy patients can consistently use and benefit from wearable devices: device cost and practicality, comfort of wearing the device, personal preferences, visualization and accuracy of monitoring data [55].While disease-related factors do not necessarily impact the adoption of digital technology [56], patient characteristics such as age and income may influence preferences and serve as potential barriers to use [57].

### Proper usage of wearable devices

The primary goal of seizure detection wearables is to enhance safety before and after seizures. Some users may assume that they only need to wear the device while sleeping. However, 30% of SUDEP cases occur while patients are awake [10]. Given the unpredictability of seizures, occasional non-use of wearable DHT devices during daytime or nighttime could reduce their potential benefits. For DHT devices to be effective, continuous wearing is essential. Ensuring that users are informed about correct usage practices is crucial for maximizing their impact.

## Limitations of using digital services and devices: a critical appraisal

To provide a rigorous and structured analysis of the obstacles, this section synthesizes the limitations discussed throughout the manuscript into a focused critical appraisal, referencing specific studies and their findings.

### The quality of recorded data

Despite advancements in wearable devices and mobile health technologies for seizure detection and prediction, these technologies still lack sufficient clinical reliability and practicality in optimizing epilepsy management and reducing related morbidity and mortality [38]. One of the key challenges is the quality of recorded data [58, 59]. The transient nature of seizures requires high time-resolution monitoring, while existing biosensors rely on machine learning to quantify signal quality and automatically filter out low-quality data [60]. The autonomous classification of wearable biosensor signals using machine learning must include quantitative assessments of signal quality to reject damaged or low-quality segments automatically. Current wearable devices still require further development to improve data quality, consistency management, and user acceptability [61].

### Accuracy and timing of seizure detection

The accuracy and timing of seizure detection are critical since false positives and misdiagnoses can negatively impact patients, often leading to device discontinuation [62]. Studies indicate that achieving a detection sensitivity of over 90% is crucial, though the acceptable false alarm rate varies depending on seizure frequency [57]. Current wearable devices have high false alarm rates, primarily detecting convulsive or motor seizures, while detection for other seizure types remains under development [34, 63, 64]. When used in combination with seizure diaries, wearable devices may enhance the accuracy and completeness of a patient's seizure history [23]. However, data supporting the ability of wearable DHT to reduce injury risk is limited, and there is no evidence that they can lower the risk of SUDEP. Additionally, there is currently no confirmed evidence that using these devices can quantify seizures or improve treatment outcomes.

### Impact on health-related quality of life

While wearable devices have the potential to reduce seizure-related anxiety, thereby improving health-related quality of life (HR-QOL), the actual improvement is often limited. Seizure detection devices may enhance seizure safety, reduce caregivers' hyper-vigilance, and lower seizure-related anxiety, contributing to moderate or significant anxiety relief for many epilepsy patients and caregivers [57]. However, their overall impact on HR-QOL remains limited. Data security, privacy protection, and compatibility with optimal software and hardware are also key concerns for users [65]. The FDA has established cybersecurity requirements for digital platforms and medical-use devices [66], highlighting the importance of security measures in this field.

### Battery life and cost considerations

Battery life and device cost are also important considerations. Ideally, the device should be worn continuously during both sleep and wakefulness, but frequent recharging may impact user adherence [56]. While FDA-approved devices may be covered by insurance, the high out-of-pocket costs remain a significant barrier to widespread adoption [67].

In summary, while wearable devices show great potential in epilepsy management, they still face multiple challenges related to data accuracy, user acceptance, and economic feasibility. These factors need to be carefully considered in future product design and policy development to enhance their effectiveness and accessibility.

## Future needs and challenges of wearable devices for epilepsy

The acceptability of wearable seizure detection devices is the primary consideration for their long-term use. While these devices can identify tonic–clonic seizures, detecting other types of seizures remains a challenge. Studies indicate that combining long-term wearable device data with electronic seizure diaries can effectively predict seizure risk probabilities [23]. The design, comfort, and detection performance of these devices are key factors influencing their sustained use. The impact of wearable health technologies has been widely studied across various medical processes and disease management, showing positive, negative, or neutral effects [68]. Systematic reviews suggest that addressing key technical support components, enhancing data security, improving digital literacy, and optimizing operational standards can significantly reduce current barriers to the adoption and accessibility of DHT [69, 70]. Future advancements in technology may integrate clinical records with data from wearable devices and mobile applications, allowing for a deeper analysis of patient-specific risk factors. Cloud computing could be leveraged to incorporate these factors into personalized epilepsy risk prediction models.

Seizure prediction is a dynamic and evolving field. Many epilepsy patients consider the unpredictability of seizures as one of the most distressing aspects of disease management [71]. The complexity of seizure prediction goes beyond simple seizure detection. Wearable devices show potential in utilizing biomarkers from seizure and activity cycles for patient-specific predictions. This capability may allow patients to develop safety plans, adjust daily activities, or take emergency medical precautions before an impending seizure [46]. One of the emerging research areas is brain-computer interfaces (BCIs), which are increasingly being explored for treating epilepsy, depression, and movement disorders [72, 73]. EEG-based BCI wearable devices have already been applied in seizure prediction [74].

A critical area of current research is the effective presentation of DHT data to healthcare professionals and patients, ensuring data accuracy and reliability for clinical decision-making [18]. In recent years, new wearable and portable EEG solutions have been developed for both short-term and long-term monitoring of epilepsy patients. The application of wireless and Bluetooth EEG aims to overcome the limitations of conventional EEG and improve the usability of these devices [75, 76]. However, these innovative solutions have not yet been widely adopted for routine monitoring of epilepsy patients. Artificial intelligence (AI) holds significant potential for improving the diagnosis and management of epilepsy. While early applications of AI in epilepsy have been predominantly driven by machine learning, deep learning approaches are emerging as a promising direction for future development. Video recordings combined with AI have already been utilized for seizure detection and seizure classification [77, 78]. Despite the development of numerous AI tools with potential for epilepsy diagnosis and management, few have been implemented in clinical practice.

The potential of AI in epilepsy management is immense, offering the prospect of transformative, patient-centered care and predictive interventions. In the future, AI systems may be capable of predicting seizures hours or even days in advance by analyzing large volumes of clinical data, genetic predispositions, and environmental factors. These systems could be seamlessly integrated into daily life, continuously monitoring patients through wearable or even permanently implanted devices, alerting them to potential triggers. They may even respond in real time by modulating neural activity or delivering medications directly to specific brain regions via neurostimulation or automated drug release mechanisms [79]. At the same time, with the increasing use of artificial intelligence [80, 81], ensuring patient data security and privacy has become a critical issue that cannot be overlooked [82, 83].

In conclusion, wearable devices offer promising opportunities for improving epilepsy management by enhancing patient autonomy and engagement [82]. However, challenges remain regarding their usability, acceptability, and integration within existing healthcare systems. Addressing these issues is essential for realizing the full potential of wearable technologies, with significant implications for patients, clinicians, and researchers.

## Conclusions

Wearable digital health technologies demonstrate immense potential in epilepsy management, not only by enhancing patients' control over their condition but also by enabling early seizure detection, prediction, and personalized intervention. These technologies support more precise health monitoring and data analysis, helping both patients and healthcare providers better manage the unpredictability of epilepsy and improve overall well-being. However, digital health promotion strategies often overemphasize individual responsibility, overlooking the broader social, cultural, and ethical considerations associated with using these technologies. Therefore, when developing and deploying these devices, it is crucial to ensure data validity, cybersecurity, and privacy protection, promote equitable access, and comprehensively address potential risks and ethical concerns arising from the integration of artificial intelligence.

To enhance epilepsy management comprehensively, future advancements should focus on improving multimodal sensor technologies, integrating clinical records with existing device data, and expanding the detection capabilities to a wider range of epilepsy types. Through these efforts, wearable digital health technologies will better serve existing patients, contribute significant benefits to the healthcare system, and ultimately improve patient safety, quality of life, and dignity.

## Data Availability

Not applicable.

## Abbreviations

ACM: Accelerometers
AI: Artificial intelligence
BCI: Brain-computer interface
DHT: Digital health technologies
ECG: Electrocardiogram
EDA: Electrodermal activity
EEG: Electroencephalogram
EMG: Electromyography
FAR: False alarm rate
FBTCS: Focal-to-bilateral tonic–clonic seizures
GPS: Global positioning system
GTCS: Generalized tonic–clonic seizures
HR-QOL: Health-related quality of life
IFCN: International Federation of Clinical Neurophysiology
ILAE: International League Against Epilepsy
PNES: Psychogenic non-epileptic seizures
PPG: Photoplethysmography
SUDEP: Sudden unexpected death in epilepsy
